# Neuroendocrine Whiplash: Slamming the Breaks on Anabolic-Androgenic Steroids Following Repetitive Mild Traumatic Brain Injury in Rats May Worsen Outcomes

**DOI:** 10.3389/fneur.2019.00481

**Published:** 2019-05-08

**Authors:** Jason Tabor, Reid Collins, Chantel T. Debert, Sandy R. Shultz, Richelle Mychasiuk

**Affiliations:** ^1^Department of Psychology, University of Calgary, Calgary, AB, Canada; ^2^Alberta Children's Hospital Research Institute, University of Calgary, Calgary, AB, Canada; ^3^Division of Physical Medicine and Rehabilitation, Department of Clinical Neurosciences, University of Calgary, Calgary, AB, Canada; ^4^Hotchkiss Brain Institute, University of Calgary, Calgary, AB, Canada; ^5^Department of Neuroscience, Monash University, Melbourne, VIC, Australia; ^6^Department of Medicine, University of Melbourne, Melbourne, VIC, Australia

**Keywords:** concussion, adolescent, prefrontal cortex, pituitary, amygdala, HPA axis

## Abstract

Sport-related concussion is an increasingly common injury among adolescents, with repetitive mild traumatic brain injuries (RmTBI) being a significant risk factor for long-term neurobiological and psychological consequences. It is not uncommon for younger professional athletes to consume anabolic-androgenic steroids (AAS) in an attempt to enhance their performance, subjecting their hormonally sensitive brains to potential impairment during neurodevelopment. Furthermore, RmTBI produces acute neuroendocrine dysfunction, specifically in the anterior pituitary, disrupting the hypothalamic-pituitary adrenal axis, lowering cortisol secretion that is needed to appropriately respond to injury. Some AAS users exhibit worse symptoms post-RmTBI if they quit their steroid regime. We sought to examine the pathophysiological outcomes associated with the abrupt cessation of the commonly abused AAS, Metandienone (Met) on RmTBI outcomes in rats. Prior to injury, adolescent male rats received either Met or placebo, and exercise. Rats were then administered RmTBIs or sham injuries, followed by steroid and exercise cessation (SEC) or continued treatment. A behavioral battery was conducted to measure outcomes consistent with clinical representations of post-concussion syndrome and chronic AAS exposure, followed by analysis of serum hormone levels, and qRT-PCR for mRNA expression and telomere length. RmTBI increased loss of consciousness and anxiety-like behavior, while also impairing balance and short-term working memory. SEC induced hyperactivity while Met treatment alone increased depressive-like behavior. There were cumulative effects whereby RmTBI and SEC exacerbated anxiety and short-term memory outcomes. mRNA expression in the prefrontal cortex, amygdala, hippocampus, and pituitary were modified in response to Met and SEC. Analysis of telomere length revealed the negative impact of SEC while Met and SEC produced changes in serum levels of testosterone and corticosterone. We identified robust changes in mRNA to serotonergic circuitry, neuroinflammation, and an enhanced stress response. Interestingly, Met treatment promoted glucocorticoid secretion after injury, suggesting that maintained AAS may be more beneficial than abstaining after mTBI.

## Introduction

Mild traumatic brain injury (mTBI), or concussion, is a common form of head injury occurring mostly via falls, vehicular accidents, or by sports-related impacts to the head (1). mTBI imposes accelerative and rotational forces on the brain inside the skull that can result in structural, cellular, and mRNA expression changes (2), which are particularly disruptive during childhood and adolescence when the brain is still developing (3). While most people fully recover, a significant portion of those subjected to repetitive mTBIs (RmTBI), go on to exhibit clinical symptoms of post-concussive syndrome (PCS) (4). These symptoms include deficits in motor function, changes in mood and anxiety levels, as well as problems with complex mental processes and detriments to cognitive abilities (5). These symptoms may be brought about by the neurometabolic cascade that happens post-concussion (6). Following mTBI, the damaged cell membranes release excitatory neurotransmitters and cause ionic imbalances, contributing to a spread of depolarization and ionic flux in the brain (6, 7). Membrane ion pumps work exhaustively in attempts to restore ionic balance, requiring increased cellular ATP (6). The hyperglycolysis, and increased mitochondrial activity used to meet these energy demands is followed by a state of impaired metabolism, possibly contributing to behavioral impairments (7). mTBI has been shown to induce mitochondrial metabolic and genetic changes (8), in addition to mitochondrial dysfunction (9) associated with detrimental downstream effects such as axonal damage, neuron death, and a compromised blood-brain barrier (10). Children and adolescents are at highest risk for RmTBI (11), with adolescent athletes being a particularly vulnerable population (12). Given the long-term neurobiological and psychological consequences of RmTBI, and the increasing incidence of sport-related concussions in youth, understanding the pathology of RmTBI during this developmentally sensitive stage of brain maturation is crucial to predicting outcomes.

Further complicating this problem, it is not uncommon for adolescent athletes to consume anabolic-androgenic steroids (AAS) (13). AAS are a group of hormones including naturally produced testosterone and lab-made derivatives (14). Clinically they are used to treat various medical conditions, however they are also illicitly used to boost athletic performance (13). Global data indicate the prevalence of AAS use to be ~3.3%, with adolescents having higher rates of AAS use than adults (13). AAS exposure during puberty has a wide range of neurological consequences that have the potential to affect brain development. Puberty is a hormonally sensitive period where the addition of exogenous hormones can have detrimental behavioral and neurohormonal effects (15, 16). Symptoms of chronic AAS use include varying levels of anxiety (17) and depression (18), as well as increased aggression, which may persist even after individuals stop taking the drug (19).

What's more, acute neuroendocrine dysfunction has been identified after mTBI, specifically in the anterior pituitary, disrupting normal function of the hypothalamic-pituitary-adrenal (HPA) axis (20). Elevated cortisol levels after injury are beneficial and help the body protect itself by promoting healing mechanisms (21). However, following RmTBI there is often a decrease in cortisol due to this HPA axis disruption which likely contributes to worse symptomology and increased recovery time (22). Interestingly, athletes who abuse AAS, and experience RmTBI often opt to abstain from their drug and exercise regimes in an effort to expedite recovery. Like those experiencing withdrawal due to their AAS dependence, some athletes have reported worse outcomes after abstaining from AAS (23, 24) suggesting that AAS maybe providing neuroprotective, or possibly therapeutic effects on the injured brain. Despite the prevalence of AAS, relatively few studies have looked at the interaction between AAS and RmTBI in adolescents, and to our knowledge, none have investigated whether quitting AAS following an mTBI is the best choice.

Given that adolescent athletes are at particularly high risk for RmTBI and have a high rate of AAS use, we sought to investigate the pathophysiological outcomes associated with the abrupt cessation of both exercise and the commonly abused AAS, Metandienone (25) (Met) on RmTBI outcomes using a clinically relevant rodent model of concussion. Adolescent males are consistently reported with higher AAS usage than females (26–28), in addition to higher lifetime prevalence rates of AAS use (6.4%) over females (1.6%) (13), which is why they were the selected for this study. Male rats received either Met or placebo and were then randomly assigned to the RmTBI or sham injury group. Following the RmTBIs or sham injuries, half the rats were returned to normal drinking water and were deprived of exercise wheels, while the other half continued to exercise and receive Met. A behavioral test battery was conducted to measure outcomes consistent with clinical representations of PCS and chronic AAS exposure. Neurobiological outcomes were further examined via changes in mRNA expression in the prefrontal cortex (PFC), the amygdala (AMYG), the hippocampus (HPC), and the pituitary gland (PIT). The PFC, HPC, and AMYG were selected because both concussion and AAS affect executive function, short-term working memory, mood regulation, and impulsivity, which all involve neural circuitry in these 3 brain regions. The PIT was also selected for investigation as it has a role in normal hormonal function, which may be disrupted by AAS and RmTBI.

## Methods

### Subjects

All reported experiments were carried out in accordance with the Canadian Council of Animal Care and received approval from the University of Calgary Conjoint Faculties Research Ethics Approval Board. Forty-seven male Sprague Dawley rats (Charles Rivers Laboratories) were weaned at postnatal day 21 (P21), caged in groups of 4, and housed in an animal husbandry room at 21°C with a 12:12 hr light:dark cycle (lights on at 7 a.m.). The animals had *ad libitum* access to food and water.

### Exercise Protocol

At postnatal day (P) 34, rats were randomly assigned to one of the following conditions: (a) *Placebo* + *Exercise* (*n* = 8), (b) *Steroid* + *Exercise* (*n* = 8), (c) *Placebo* + *Exercise* + *Steroid and Exercise Cessation (SEC)* (*n* = 15), (d) *Steroid* + *Exercise* + *SEC* (*n* = 16). The *Exercise* groups were housed in Lafayette Activity Living Chambers (model + 80859; Lafayette, IN, USA). *SEC* groups were returned to normal cages after the third mTBI or sham injury (P46) and were deprived of Met and running wheels for the remainder of the experiment. The Activity Living Chambers were maintained in the same husbandry room as the control cages and all animals had *ad libitum* access to food and water. Activity wheel counters were used to measure the distance the rats had run, and were recorded each day.

### AAS Administration Protocol

Metandienone (Met) purchased from TripleBond (Guelph, ON, Canada) was orally administered to the rats in their drinking water by dissolving the drug at a concentration of 1.5 mg/kg, body weight/day, starting at P21. This dosage was selected as it closely mimics the dosage commonly used by humans (29) and was orally administered as this is the ingestion route most commonly observed in clinical populations. Met or placebo was administered daily to all *Steroid* and *Placebo* groups for 7 weeks up until sacrifice with the amount of water consumed was measured on a daily basis. The *SEC* groups were switched to placebo water after their third mTBI or sham injury for the remainder of the experiment (P47).

### RmTBI Procedure

At P41, rats in each group were randomly assigned to receive 3 mTBIs with the Lateral Impact (LI) device or 3 sham injuries. The LI technique employed a protocol described by Mychasiuk et al. (2). Briefly, animals were lightly anesthetized with isofluorane until a toe pinch drew no response and were then placed in a prone position on a low friction Teflon board. A 50 g weight was pneumatically fired toward the rat's head at an average speed of 8.95 ± 0.12 m/s, resulting in TBIs at 81.66 Gs. The weight impacted a small “helmet” that protected the skull from structural damage but propelled the rat into a horizontal 180° rotation. This LI technique subjects the brain to acceleration/deceleration and rotational forces that mimic a sports-related concussion (2, 30). mTBIs or sham injuries were performed on P41, P44, and P47. *Time-to-right* measured the time each rat took to wake and move from a supine position to a prone or standing position following the injury, and was used as a surrogate measure for loss-of-consciousness.

### Behavioral Testing

Rats underwent a behavioral test battery consisting of 6 behavioral tasks designed to measure post-concussive symptomology (31, 32). *Beam walking* is a test for balance and motor coordination used to measure hindleg foot slips on a tapered beam described in detail by Schallert et al. (33). This test was carried out at post-injury day 1 (PID1) and PID3.

On P49 or PID2 rats were tested in an *Open Field* paradigm as a measure of general locomotor activity (34). Animals were placed in the center of a circular arena with a diameter of 135 cm and allowed to explore their surroundings for 10 min. An over-head camera equipped with Noldus Ethovision XT 10.0 software tracked and analyzed the distance traveled and the time spent in the center of the arena for each rat. The arena was cleaned with Virkon between each testing session.

At PID3 (P50), rats were tested for general anxiety in the *Elevated Plus Maze* (EPM) (34). The EPM is constructed from black Plexiglas and was elevated 55 cm above ground. It contained two closed and two open arms, and each rat was permitted to explore the EPM for 5 min in a videotaped session. A research analyst blinded to the experimental conditions recorded how much time the rat spent in the center, open, and closed arms.

*Novel Context Mismatch* (NCM) was used to measure short term working memory. Training for the NCM occurred on P54–P56, where rats were placed in both Context A and Context B (5 min each) per training day, where Context B placement immediately followed Context A. Context A was a transparent rectangular box (70 × 40 × 33 cm) with 2 identical plastic cylinders; Context B was an opaque blue circular bin (36 cm high with a diameter of 47 cm), with two identical glass bottles. The probe trial for the test occured at PID10 (P57). Rats went from Context A (5 min) to Context B (5 min), into their home cage (5 min), then to the Novel Context (5 min). The novel context was a modified Context A, where the same rectangular box was used, and one object from Context A, as well as one object from Context B. The rats were videotaped exploring the novel context and a research assistant (blinded to the experimental conditions) scored the amount of time the animals investigated the novel object and the old object. All containers and objects were sanitized with Virkon between each testing session. The protocol employed was similar to that described be Spanswick & Sutherland (35).

Aggression levels were measured using the *Dominance Tube* test (36), which was administered on PID4. Rats were released into opposing ends of a clear tube, narrow enough to impede the animal's ability to turn around. The rats met in the middle of the tube, and the dominant animal would exhibit more aggression by forcing their opponent to back out of the tube. The rat was declared the winner when its opponent had all four paws out of the tube. The match ups at consisted of *RmTBI* vs. *RmTBI* & *Sham* vs*. Sham* rats, and were always *Steroid* vs. *Placebo* rats. There was a total of 3 trials per match up with trail wins, win percentage, and time spent in the tube recorded for each animal.

Finally, on PID14 rats were tested for 7 min in a modified *Forced Swim* paradigm similar to that employed by Yadid et al. (37) to examine depressive- or anxiety-like behaviors. A cylindrical tank (diameter of 30 cm, 60 cm high) filled with water (~25°C) high enough that the rat's tail was unable to contact the bottom of the tank. After each session, the rats were dried with warm towels and returned to their home cages with the water being replaced before the next session. The amount of time spent immobile and number of escape attempts was scored for each rat.

Experimental duration and timepoints (P41–P61) were chosen as they are reflective of adolescence in rats (38, 39).

### mRNA Analysis

Rats were euthanized at PID15 after completion of all behavioral testing. All rats were anesthetized via isoflurane inhalation, were quickly weighed and measured, and were then decapitated. Using the Zilles atlas (40) tissue from the PFC, HPC, AMYG, and PIT was removed, flash frozen on dry ice, and stored at −80°C. Total RNA was extracted from each brain region for molecular analysis with the Allprep RNA/DNA Mini Kit according to manufacturer protocols (Qiagen, Germany). Samples were tested for purity and concentration with a NanoDrop 2000 (Thermo Fisher Scientific, USA). Purified RNA (2 μg) was reverse transcribed into cDNA using the oligo(dT)20Superscript III First-Strand Synthesis Supermix Kit (Invitrogen, USA) according to manufacturer instructions.

A total of 6 genes were selected for analysis, which provided key information regarding the effects of AAS, RmTBI, and exercise on neuroinflammation, repair processes, and neurodevelopment. The 4 genes selected for PFC, AMYG, and HPC were: *Bdnf*, Brain-derived neurotrophic factor; *GR*, Glucocoritcoid receptor; *Iba1*, Ionized calcium-binding adaptor molecule 1; and *Maoa*, Monoamine oxidase A. A total of 6 genes were selected for analysis in PIT: *Bdnf*, *GR, Iba1, Maoa*, cAMP response-element binding protein (*Creb*), and Estrogen receptor (*ER*). *Bdnf* is involved in supporting the developing nervous system, with key roles in neurogenesis, neural plasticity, learning and memory (41), and is susceptible to changes in expression from mTBI (42). *GR* is a transcription factor activated by the stress hormone, cortisol (43). Under periods of injury or stress, sustained *GR* activation is toxic to neurons through increased excitotoxicity and oxidative stress (43, 44). *Iba1* is a marker of microglial activation as part of the neuroinflammatory response (45), involved in proliferation, migration, and immune responses at the site of injury (46). *Maoa* is an enzyme responsible for breaking down monoamine neurotransmitters (47) and has been implicated in the modulation of aggressive behavior (47) and stress responses via its effects on serotonergic circuitry (48, 49). *Creb* is a transcription factor that mediates complex learning and memory processes (50, 51) which can be disrupted through brain injury (52). *ER* is present throughout multiple brain regions, allowing estrogens to induce many effects on neuroprotection, synaptogenesis, and cognitive function (53).

Primers for the qRT-PCR were designed by an in-house research technician using Primer3 (http://bioinfo.ut.ee/primer3), and purchased from Integrated DNA Technologies (Coralville, USA). Duplicate samples were run on a 96-well-plate and each target gene was processed. qRT-PCR was performed and analyzed with the Applied Biosystems™ StepOnePlus™ Real-Time PCR System (Thermo Fisher Scientific, USA) with 10 ng of cDNA, 10 μM of the forward and reverse primers for each target gene, and 1X SYBR Green FastMix with Rox (Quanta Biosciences, USA). Two housekeeping genes, CycA and Ywhaz (54) were used to determine relative target gene expression through the 2^ΔΔCt^ method as previously described by Pfaffl (55).

### Telomere Length Analysis

Immediately after each rat was euthanized a sample of ear notch tissue was taken from each rat and stored at −80°C. Extraction of genomic DNA from ear notch samples using the Sigma REDExtract N-AMP Tissue PCR kit was performed according to manufacturer's protocol. Concentration and quality were measured using the NanoDrop 2000 (Thermo Fisher Scientific, USA). Samples were diluted to concentrations of 20 ng/μL to perform telomere analysis. A research technician designed primers for telomeres as well as the single copy 36B4 gene in-house with information previously described by Cawthorn (56). PCR reactions were run in duplicates on a 96 well-plate. Each PCR reaction was comprised of 1 μL of gDNA within a total volume of 20 μL using 1X SYBR Green FastMix with Rox (Quanta Biosciences, USA) for qRT-PCR on a CFX Connect Real Time PCR Detection System (Bio-Rad, Hercules, USA). The concentrations for primers were 20 μM for the forward and reverse primers of both Tel and 36B4. The telomere repeat number to single copy ratio (T/S) was calculated to determine telomere length with the single copy corresponding to the 36B4 gene. When T/S ratios = 1, the unknown DNA is equivalent to the reference DNA. If T/S > 1 there is an increase in telomere repeat number, whereas when T/S < 1, there is a decrease in telomere repeat number. T/S ratio was determined to be [2^Ct(telomere)^/2^Ct(36B4)^]– 1 = – 2^ΔCt^. Relative telomere length was determined using a linear regression equation, y = 1910.5x + 4157 (where y = telomere length and x = – 2^ΔCt^), described by Cawthorn (56).

### Serum Biomarker Analysis

Rats were euthanized via rapid decapitation on PID15, and trunk blood was collected in serum separator tubes. Samples were clotted for 30 min at room temperature then centrifuged at 1,000 g for 15 min. The serum was stored at −80°C. ELISA kits were purchased for Testosterone, Corticosterone, and Alanine Transaminase (Abcam Inc, Canada). ELISAs for each biomarker were completed according to manufacturer's instructions. Standards, positive and negative controls, and samples were all run in duplicate on a 96-well plate and measured using the BioTek Synergy H.T. plate reader and Gen5 2.00.18 software with a path length correction algorithm. Samples were all in normal range of the standard curve.

### Statistical Analysis

A research analyst blinded to all experimental conditions scored each of the behavioral tests and two analysts performed the qRT-PCR analysis for each gene, carried out in duplicate. A research analyst also carried out telomere and serum biomarker analysis. All analyses were performed with SPSS 23.0 for Mac, and *p* ≤ 0.05 was considered statistically significant. Three-way ANOVAs with Injury (RmTBI vs. Sham), Treatment (Met vs. Placebo), and SEC (cessation vs. maintenance) as factors were run for each of the behavioral and molecular outcomes. *Post-hoc* pairwise comparisons (LSD), were carried out when appropriate. All error bars on graphs represent ± SEM.

## Results

### Animal Characteristics

Weight gain from the initial mTBI to the end of the study (P41–P61), in addition to brain weight at time of sacrifice was recorded. The three-way ANOVA of body weight gain demonstrated a main effect of SEC [*F*_(1, 78)_ = 13.18, *p* < 0.01], but not of RmTBI or treatment. Interestingly, steroid treatment alone had no effect on change in body weight, but cessation of treatment and exercise significantly increased the amount of weight gained between TBI and sacrifice (Steroid + SEC cessation; 125.9 g ± 4.9; Steroid + SEC Maintenance; 112.3 g ± 3.8; Placebo + SEC cessation; 123.3 g ± 4.9; Placebo + SEC Maintenance; 108.3 g ± 3.8). This result suggests that the weight gain was associated with a loss of running wheel access, rather than cessation of steroids. There were also no significant interactions between RmTBI, treatment, or SEC. Conversely, there were no significant effects of RmTBI, treatment, or SEC for brain weight, nor were there any significant interactions. There were also no significant differences in the average distance run per day for animals in the AAS and placebo groups.

### Behavioral Testing

**Figure 1:**
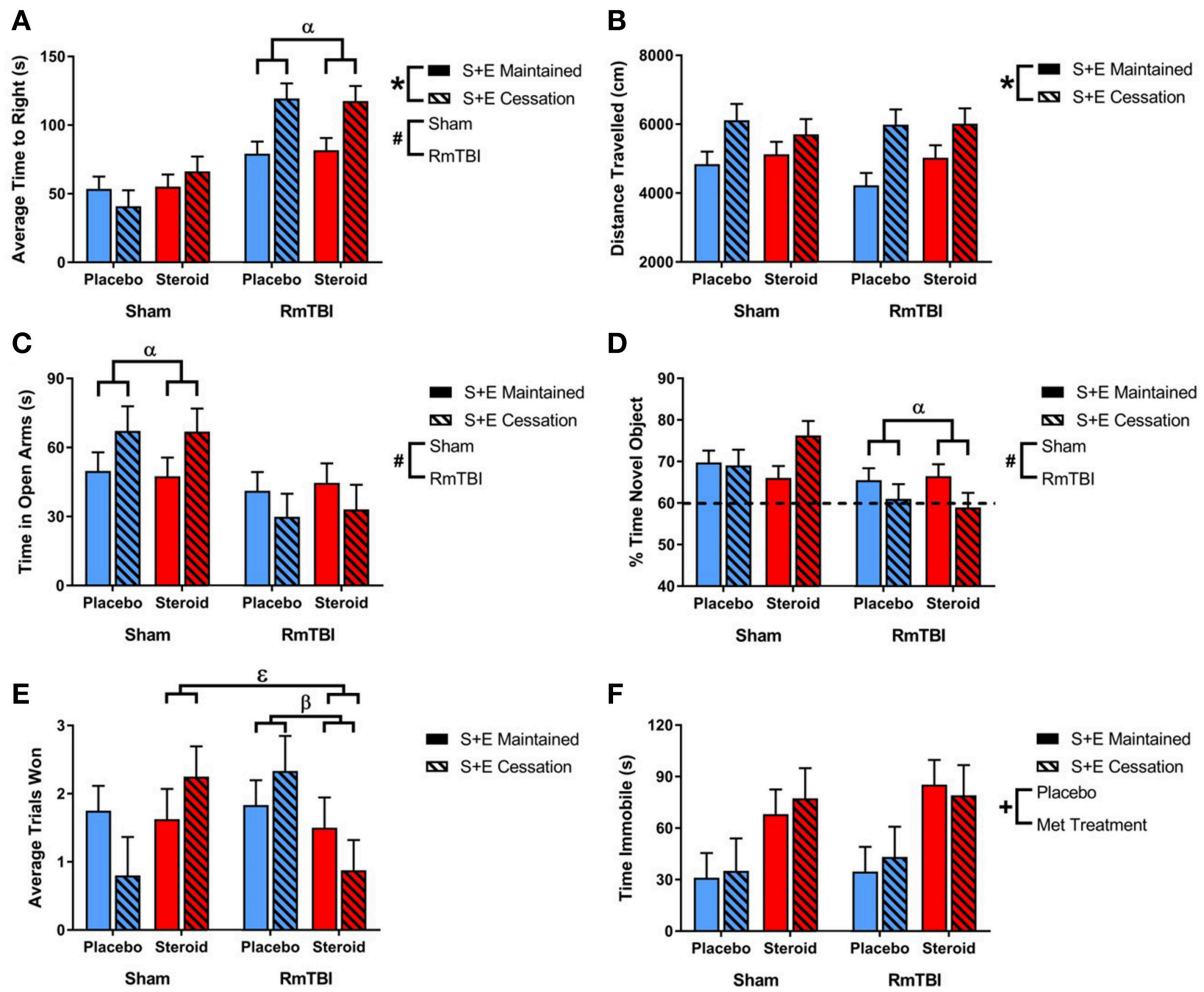
Bar graphs displaying outcomes from behavioral test battery for all groups. Means ± standard error are displayed where ^*^main effect for SEC, ^#^main effect of RmTBI, ^+^main effect of Met treatment, ^α^significant RmTBI by SEC interaction, ^β^significant Met treatment by RmTBI interaction, and ^ε^significant Met treatment by RmTBI by SEC interaction *p* ≤ 0.05. **(A)** Displays the average time-to-right after sham injury or RmTBI. *Post hoc* analysis demonstrated that the Injury × SEC interaction was driven by the animals in the RmTBI group, whereby injured animals that experienced cessation of AAS and exercise exhibited increased loss of consciousness, *p* < 0.01. **(B)** Displays the mean distance traveled in the open field test. **(C)** Displays the average time spent in the open arms of the elevated plus maze. *Post hoc* analysis of time in open arms of the EPM demonstrated that the Injury × SEC interaction was driven by the sham group, where animals that were exposed to sham injuries and experienced cessation of AAS and exercise displayed reduced anxiety-like behavior and increased time in the open arms of the EPM, *p* = 0.05. **(D)** Displays the % of time spent with a novel object in the NCM task. *Post-hoc* analysis showed that the Injury × SEC interaction was driven by the RmTBI animals, whereby injured animals that experienced cessation of AAS and exercise exhibited worse performance on the NCM task than injured animals that maintained AAS and exercise treatment, *p* < 0.01. The hatched line indicates the % of expected time the rat will spend investigating the novel object. **(E)** Displays the average trials won out of 3 possible trials in the dominance tube test. *Post hoc* analysis demonstrated that the Met Treatment by Injury interaction was driven by the RmTBI group. Animals that experienced RmTBI and placebo were more aggressive than RmTBI animals consuming AAS, *p* = 0.05. In addition, *Post hoc* analyses of the 3-way interaction between Met Treatment, Injury, and SEC found this interaction to be driven by animals in the S+E cessation group, whereby dominance/aggression increased in sham animals who experienced cessation of AAS and exercise (*p* = 0.05), but decreased in RmTBI animals that experienced cessation of AAS and exercise (*p* = 0.03). **(F)** Displays the mean time spent immobile in the forced swim test. RmTBI, repetitive mild traumatic brain injury; S+E, steroid and exercise; NCM, novel context mismatch.

All statistical results from the three-way ANOVAs for the behavioral tests can be found in Table 1 and graphical representation of these findings are in Figure 1. In summary, SEC affected behavioral performance on 2 of 7 measures (increased time-to-right and resulted in hyperactivity in the open-field) for both sham and RmTBI animals. The RmTBIs impaired performance on 4 of 7 tasks (increased time-to-right, increased hindleg foot slips in the beam walk task, and increased anxiety-like behavior in the elevated plus maze, while decreasing short-term working memory on the novel context mismatch task). AAS treatment was found to produce dysfunction in the forced swim task for both sham and RmTBI animals, whereby AAS animals displayed increased depressive-like behavior. In addition, there were multiple SEC by Injury interactions; (1) for time-to-right there was a two-way interaction between SEC and RmTBI. *Post-hoc* analysis demonstrated that the Injury × SEC interaction was driven by the animals in the RmTBI group, whereby injured animals that experienced cessation of AAS and exercise exhibited increased loss of consciousness, *p* < 0.01. (2) In the EPM there was also a two-way interaction between SEC and RmTBI. *Post-hoc* analysis of time in open arms of the EPM demonstrated that the Injury × SEC interaction was driven by the sham group, where animals that were exposed to sham injuries and experienced cessation of AAS and exercise displayed reduced anxiety-like behavior and increased time in the open arms of the EPM, *p* = 0.05. (3) In the NCM task, *post-hoc* analysis showed that the Injury × SEC interaction was driven by the RmTBI animals, whereby injured animals that experienced cessation of AAS and exercise exhibited worse performance on the NCM task than injured animals that maintained AAS and exercise treatment, *p* < 0.01. Finally, in the dominance tube task of aggression we identified a Met treatment by SEC interaction and a three-way interaction. *Post-hoc* analysis demonstrated that the Met Treatment by Injury interaction was driven by the RmTBI group. Animals that experienced RmTBI and placebo were more aggressive than RmTBI animals consuming AAS, *p* = 0.05. In addition, *post-hoc* analyses of the 3-way interaction between Met Treatment, Injury, and SEC found this interaction to be driven by animals in the S+E cessation group, whereby dominance/aggression increased in sham animals who experienced cessation of AAS and exercise (*p* = 0.05), but decreased in RmTBI animals that experienced cessation of AAS and exercise (*p* = 0.03).

**Table 1:** Statisitcal results of the three-way anovas for the behavioral assessment of rmtbi, aas treatment, and SEC in adolescent rats.

**Behavioral test**	**Effect of RmTBI F (*p*)**	**Effect of Met Treatment F (*p*)**	**Effect of SEC F (*p*)**	**Met Treatment × RmTBI F (*p*)**	**Met Treatment × SEC F (*p*)**	**RmTBI × SEC F (*p*)**	**Met Treatment × RmTBI × SEC F (*p*)**
Time-to-right	40.56 (**<**0.01)	0.93 (0.34)	6.82 (0.01)	0.85 (0.36)	0.46 (0.50)	7.42 (< 0.01)	0.98 (0.33)
Beam walk	10.52 (< 0.01)	0.03 (0.86)	0.12 (0.74)	0.24 (0.63)	2.59 (0.11)	1.62 (0.21)	0.27 (0.61)
Open field: distance	0.22 (0.64)	0.38 (0.54)	15.65 (< 0.01)	0.68 (0.41)	1.58 (0.21)	0.60 (0.44)	0.00 (0.95)
Open field: center	0.11 (0.75)	0.68 (0.41)	0.16 (0.69)	0.28 (0.60)	0.01 (0.91)	0.00 (0.97)	1.03 (0.31)
EPM	9.73 (< 0.01)	0.02 (0.88)	0.29 (0.60)	0.12 (0.73)	0.00 (0.95)	5.08 (0.03)	0.01 (0.93)
NCM	10.11 (< 0.01)	0.07 (0.80)	0.08 (0.78)	0.25 (0.62)	0.73 (0.40)	5.48 (0.02)	2.31 (0.13)
Dominance tube	0.01 (0.93)	0.13 (0.72)	0.12 (0.73)	5.88 (0.02)	0.12 (0.73)	0.02 (0.88)	4.41 (0.04)
Forced swim	0.45 (0.51)	12.98 (< 0.01)	0.11 (0.74)	0.03 (0.87)	0.04 (0.84)	0.06 (0.81)	0.19 (0.67)

### mRNA Expression

mRNA was examined for 4 different genes (*Bdnf, GR, Iba1*, and *Maoa*) in the PFC, AMYG, and HPC, and 6 different genes (*Bdnf*, *GR, Iba1, Maoa, ER*, and *Creb*) in the PIT.

#### PFC

**Figure 2:**
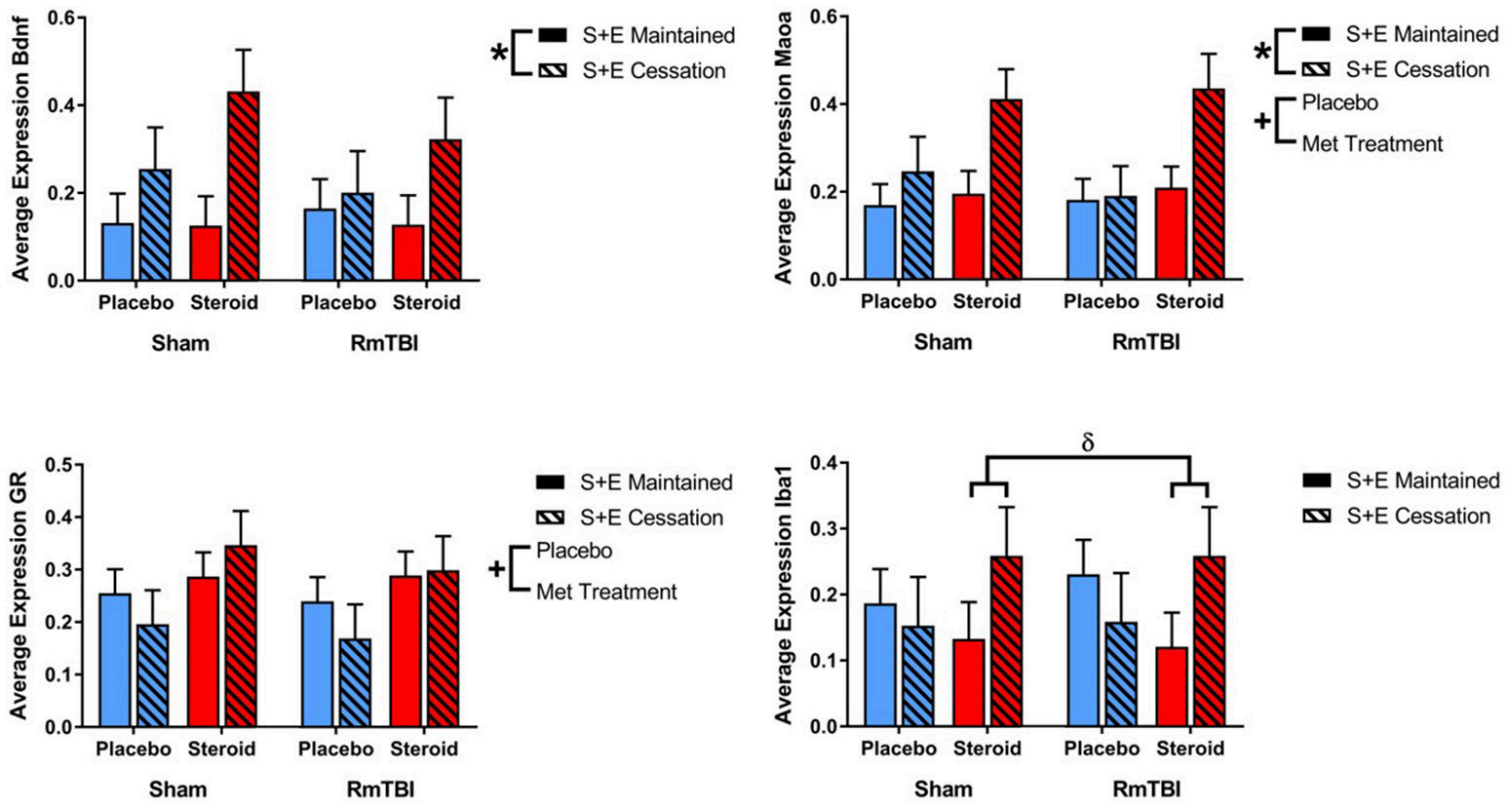
Bar graphs displaying average mRNA expression levels in the PFC. Means ± standard error are displayed where ^*^main effect for SEC, ^+^main effect of Met treatment, and ^δ^significant Met treatment by SEC interaction *p* ≤ 0.05. For *Iba1, post-hoc* analysis demonstrated that the Met Treatment by SEC interaction was driven by animals exposed to AAS. Rats that were exposed to AAS and exercise and then subsequently quit, exhibited increased expression of *Iba1*, when compared to rats that were exposed to AAS and exercise throughout the experiment, *p* = 0.04. mRNA, messenger RNA; PFC, pre-frontal cortex; RmTBI, repetitive mild traumatic brain injury; S+E, steroid and exercise.

In the PFC expression, 2 of 4 genes were influenced by Met treatment (*GR* and *Maoa*), 2 were influenced by SEC (*Bdnf*, and *Maoa*), and 1 gene (*Iba1*) exhibited a Met treatment by SEC interaction. For *Iba1, post-hoc* analysis demonstrated that the Met Treatment by SEC interaction was driven by animals exposed to AAS. Rats that were exposed to AAS and exercise and then subsequently quit, exhibited increased expression of *Iba1*, when compared to rats that were exposed to AAS and exercise throughout the experiment, *p* = 0.04 (see Figure 2).

#### AMYG

**Figure 3:**
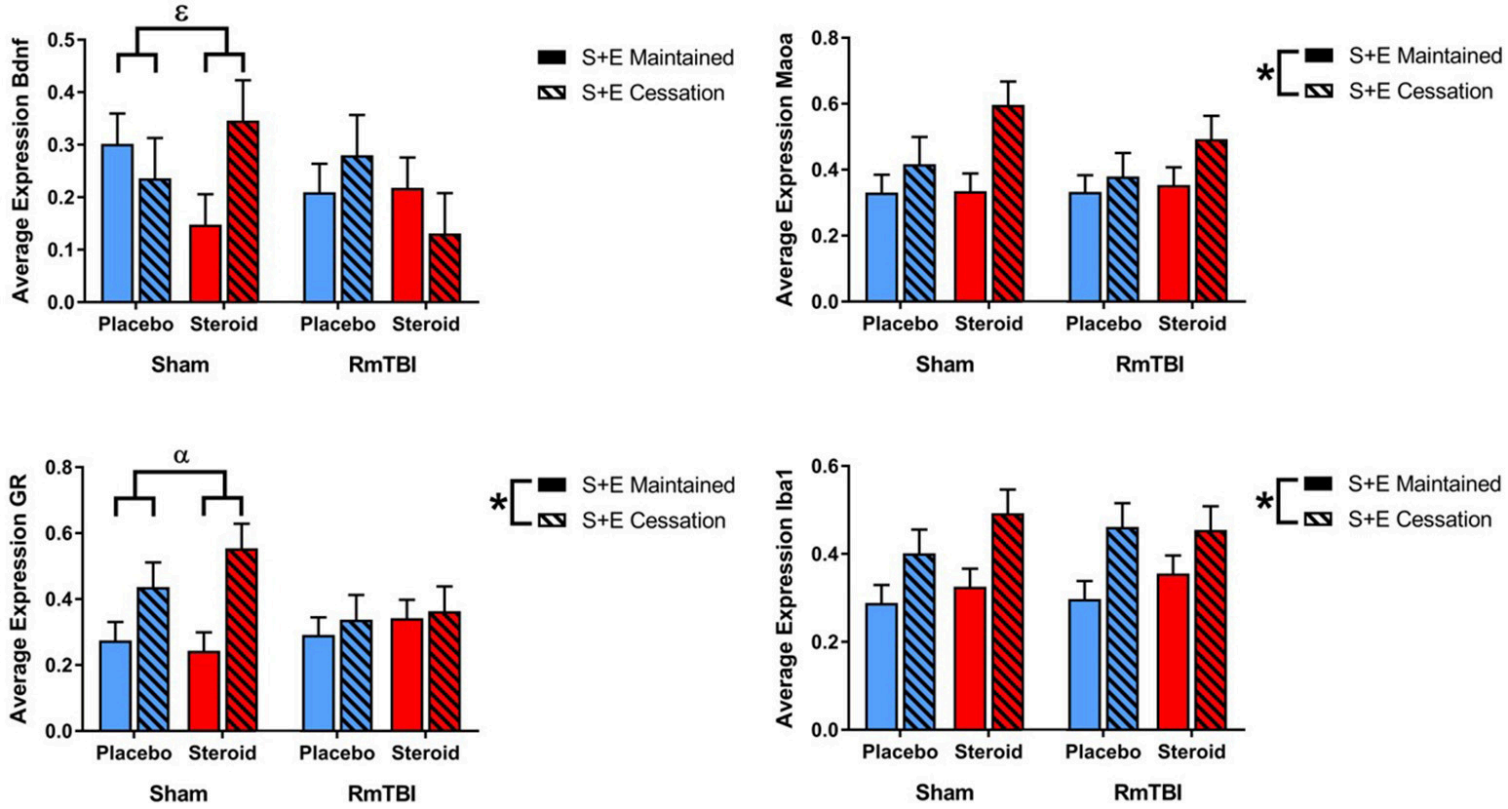
Bar graphs displaying average mRNA expression in the AMYG. Means ± standard error are displayed where ^*^main effect for SEC, ^α^significant injury by SEC interaction, and ^ε^significant Met treatment by RmTBI by SEC interaction *p* ≤ 0.05. *Post-hoc* analysis showed that the Injury by SEC interaction of *GR* was driven by the sham animals, whereby expression of *GR* was significantly increased in sham animals that experienced cessation of AAS and exercise, compared to sham animals that maintained AAS and exercise throughout the experiment, *p* < 0.01. *Post-hoc* analysis of the three-way interaction between Injury, Met Treatment and SEC of *Bdnf* demonstrated that the effect was driven by Sham animals that received AAS, whereby expression of *Bdnf* was significantly elevated in animals that experienced cessation of AAS and exercise as compared to those that maintained treatment throughout the experiment, *p* < 0.05. mRNA, messenger RNA; AMYG, amygdala; RmTBI, repetitive mild traumatic brain injury; S+E, steroid and exercise.

SEC had more influence on gene expression in the AMYG, affecting 3 of 4 genes (*GR, Iba1*, and *Maoa*). The AMYG also exhibited significant interactions; (1) *GR* demonstrated a significant RmTBI by SEC interaction. *Post-hoc* analysis showed that the Injury by SEC interaction was driven by the sham animals, whereby expression of *GR* was significantly increased in sham animals that experienced cessation of AAS and exercise, compared to sham animals that maintained AAS and exercise throughout the experiment, *p* < 0.01. (2) *Bdnf* displayed a significant three-way interaction. *Post-hoc* analysis of the three-way interaction between Injury, Met Treatment and SEC demonstrated that the effect was driven by Sham animals that received AAS, whereby expression of *Bdnf* was significantly elevated in animals that experienced cessation of AAS and exercise as compared to those that maintained treatment throughout the experiment, *p* < 0.05 (see Figure 3).

#### HPC

**Figure 4:**
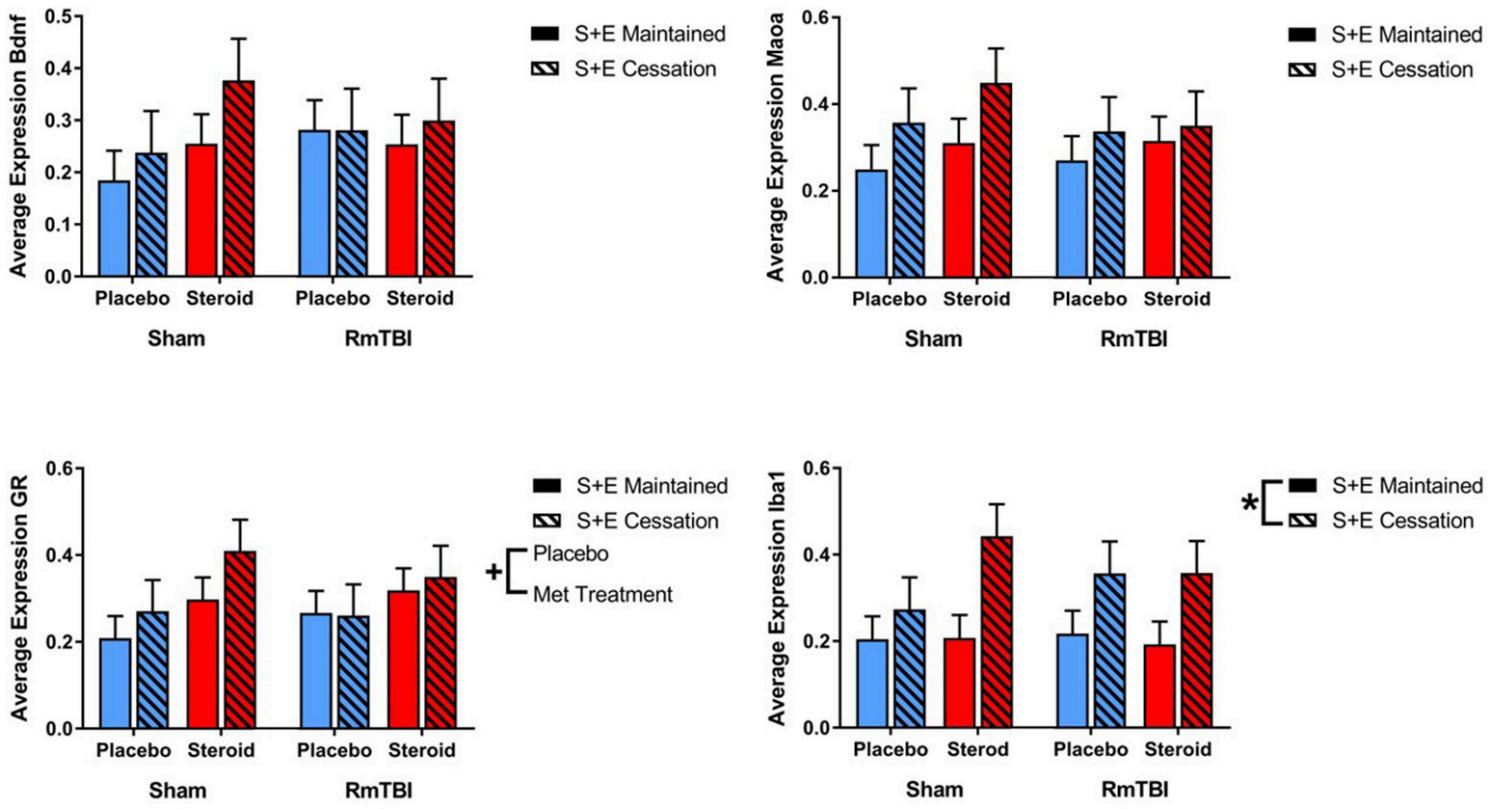
Bar graphs displaying average mRNA expression in the HPC. Means ± standard error are displayed where ^*^main effect for SEC and ^+^main effect of Met treatment *p* ≤ 0.05. mRNA, messenger RNA; HPC, hippocampus; RmTBI, repetitive mild traumatic brain injury; S+E, steroid and exercise.

Gene expression in the HPC was the least affected of the four brain regions, with only 1 gene (*GR*) altered by Met treatment, and 1 gene (*Iba1*) influenced by SEC (see Figure 4).

#### PIT

**Figure 5:**
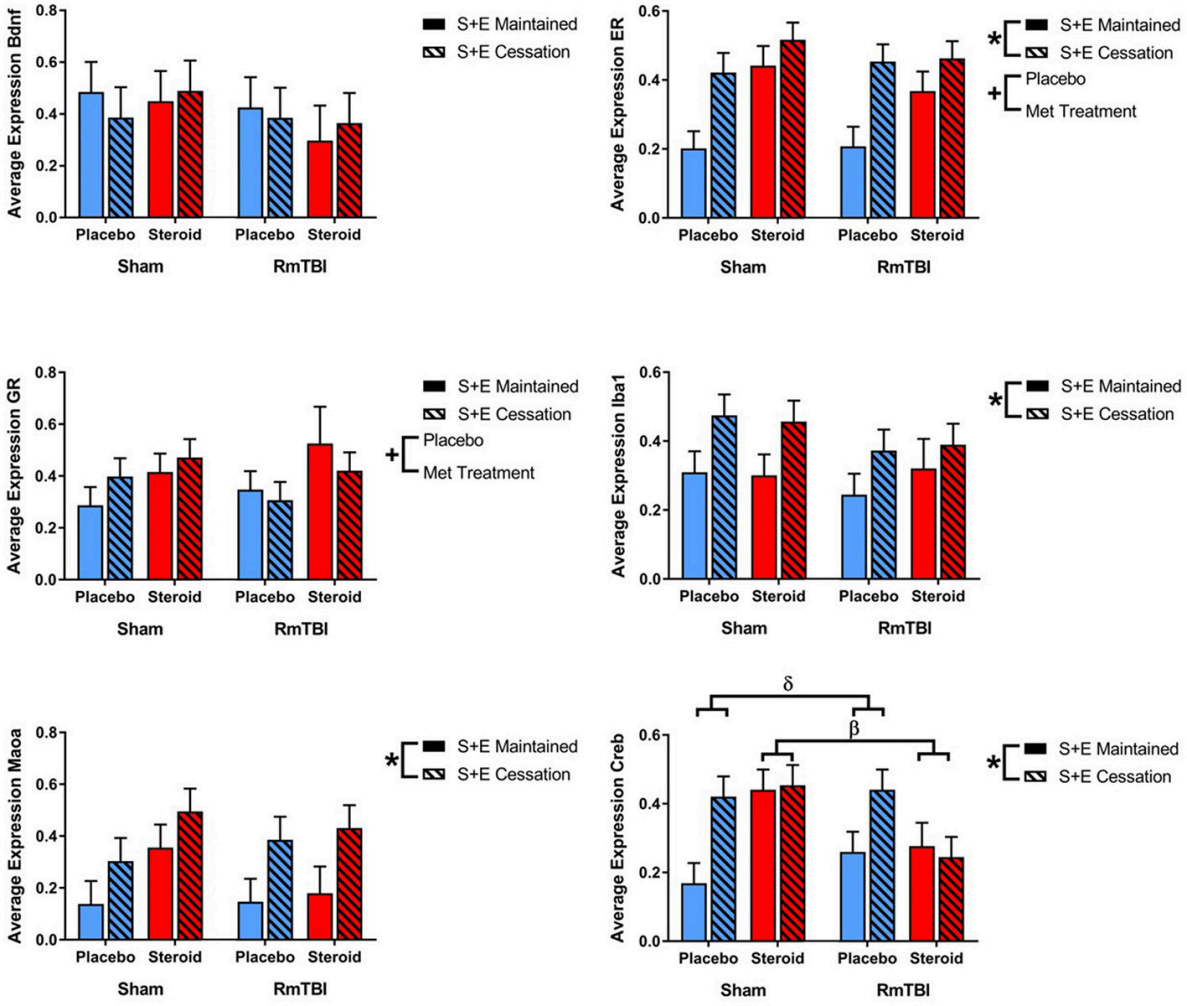
Bar graphs displaying average mRNA expression levels in the PIT. Means ± standard error are displayed where ^*^main effect for SEC, ^+^main effect of Met treatment, ^β^significant Met treatment by RmTBI interaction, and ^δ^significant Met treatment by SEC interaction *p* ≤ 0.05. *Post-hoc* analysis of *Creb* demonstrated that the Met Treatment by Injury interaction was driven by animals in the Steroid group. Rats with sham injuries in the steroid group exhibited greater expression of *Creb* than rats with RmTBI in the steroid group, *p* < 0.01. The second *post-hoc* analysis found that the Met Treatment by SEC interaction was driven by the placebo group, whereby cessation of AAS and exercise significantly increased expression of *Creb* in animals that did not receive AAS, *p* < 0.01. mRNA, messenger RNA; PIT, pituitary; RmTBI, repetitive mild traumatic brain injury; S+E, steroid and exercise.

Lastly, in the PIT, 2 out of 6 genes (*ER* and *GR*) were altered by treatment and 4 of 6 genes (*ER, Iba1, Maoa*, and *Creb*) were affected by SEC. Expression of 1 gene (Creb) exhibited a Met treatment by Injury interaction, as well as a Met treatment by SEC interaction. *Post-hoc* analysis demonstrated that the Met Treatment by Injury interaction was driven by animals in the Steroid group. Rats with sham injuries in the steroid group exhibited greater expression of *Creb* than rats with RmTBI in the steroid group, *p* < 0.01. The second *post-hoc* analysis found that the Met Treatment by SEC interaction was driven by the placebo group, whereby cessation of AAS and exercise significantly increased expression of *Creb* in animals that did not receive AAS, *p* < 0.01 (see Figure 5).

See Table 2 for summary of statistical results of genes in all four brain regions.

**Table 2:** Changes in gene expression in the pfc, amyg, hpc, and pit after rmtbi, metandienone treatment, and SEC.

**Brain region**	**Gene**	**Effect of RmTBI F (*p*)**	**Effect of met treatment F (*p*)**	**Effect of SEC F (*p*)**	**Met treatment × RmTBI F (*p*)**	**Met Treatment × SEC F (*p*)**	**RmTBI × SEC F (*p*)**	**Met Treatment × RmTBI × SEC F (*p*)**
PFC	*Bdnf*	0.30 (0.59)	1.22 (0.28)	8.02 (< 0.01)	0.13 (0.72)	2.15 (0.15)	0.72 (0.40)	0.01 (0.92)
	*GR*	0.30 (0.59)	5.16 (0.03)	0.14 (0.71)	0.00 (0.99)	1.59 (0.21)	0.15 (0.70)	0.06 (0.81)
	*Iba1*	0.04 (0.84)	0.05 (0.82)	0.74 (0.39)	0.11 (0.74)	4.10 (0.05)	0.02 (0.89)	0.08 (0.79)
	*Maoa*	0.00 (0.98)	6.89 (0.01)	8.90 (< 0.01)	0.21 (0.65)	4.02 (0.05)	0.10 (0.75)	0.19 (0.66)
AMYG	*Bdnf*	1.01 (0.32)	0.94 (0.34)	0.35 (0.56)	0.26 (0.62)	0.32 (0.58)	0.61 (0.44)	4.87 (0.03)
	*GR*	0.88 (0.35)	0.77 (0.39)	8.43 (< 0.01)	0.00 (0.95)	0.44 (0.51)	4.70 (0.04)	0.84 (0.37)
	*Iba1*	0.20 (0.66)	1.75 (0.20)	15.94 (**<**0.01)	0.32 (0.57)	0.01 (0.93)	0.02 (0.90)	0.76 (0.40)
	*Maoa*	0.44 (0.51)	3.04 (0.09)	8.53 (< 0.01)	0.07 (0.79)	2.14 (0.15)	0.78 (0.38)	0.21 (0.65)
HPC	*Bdnf*	0.10 (0.75)	1.04 (0.32)	1.25 (0.27)	1.24 (0.27)	0.36 (0.56)	0.43 (0.52)	0.01 (0.92)
	*GR*	0.00 (0.96)	4.34 (0.04)	1.26 (0.27)	0.24 (0.63)	0.23 (0.63)	0.72 (0.40)	0.01 (0.94)
	*Iba1*	0.00 (0.98)	0.65 (0.43)	11.18 (< 0.01)	1.17 (0.29)	1.12 (0.30)	0.00 (0.99)	0.59 (0.45)
	*Maoa*	0.22 (0.64)	1.19 (0.28)	3.20 (0.08)	0.24 (0.63)	0.00 (0.99)	0.56 (0.46)	0.10 (0.75)
PIT	*Bdnf*	1.01 (0.33)	0.06 (0.81)	0.01 (0.93)	0.41 (0.53)	0.54 (0.47)	0.06 (0.81)	0.01 (0.92)
	*ER*	0.35 (0.56)	11.02 (< 0.01)	17.58 (< 0.01)	1.21 (0.29)	3.77 (0.07)	0.09 (0.77)	0.00 (0.97)
	*GR*	0.02 (0.90)	4.44 (0.05)	0.01 (0.93)	0.15 (0.71)	0.262 (0.61)	1.77 (0.20)	0.00 (0.97)
	*Iba1*	1.37 (0.26)	0.13 (0.73)	7.93 (0.01)	0.42 (0.52)	0.14 (0.72)	0.46 (0.51)	0.07 (0.79)
	*Maoa*	0.34 (0.57)	3.62 (0.07)	9.61 (< 0.01)	1.67 (0.21)	0.00 (0.96)	0.52 (0.48)	0.02 (0.88)
	*Creb*	2.38 (0.14)	0.56 (0.46)	5.93 (0.02)	8.16 (0.01)	7.08 (0.01)	0.47 (0.50)	0.02 (0.88)

### Telomere Length

**Figure 6:**
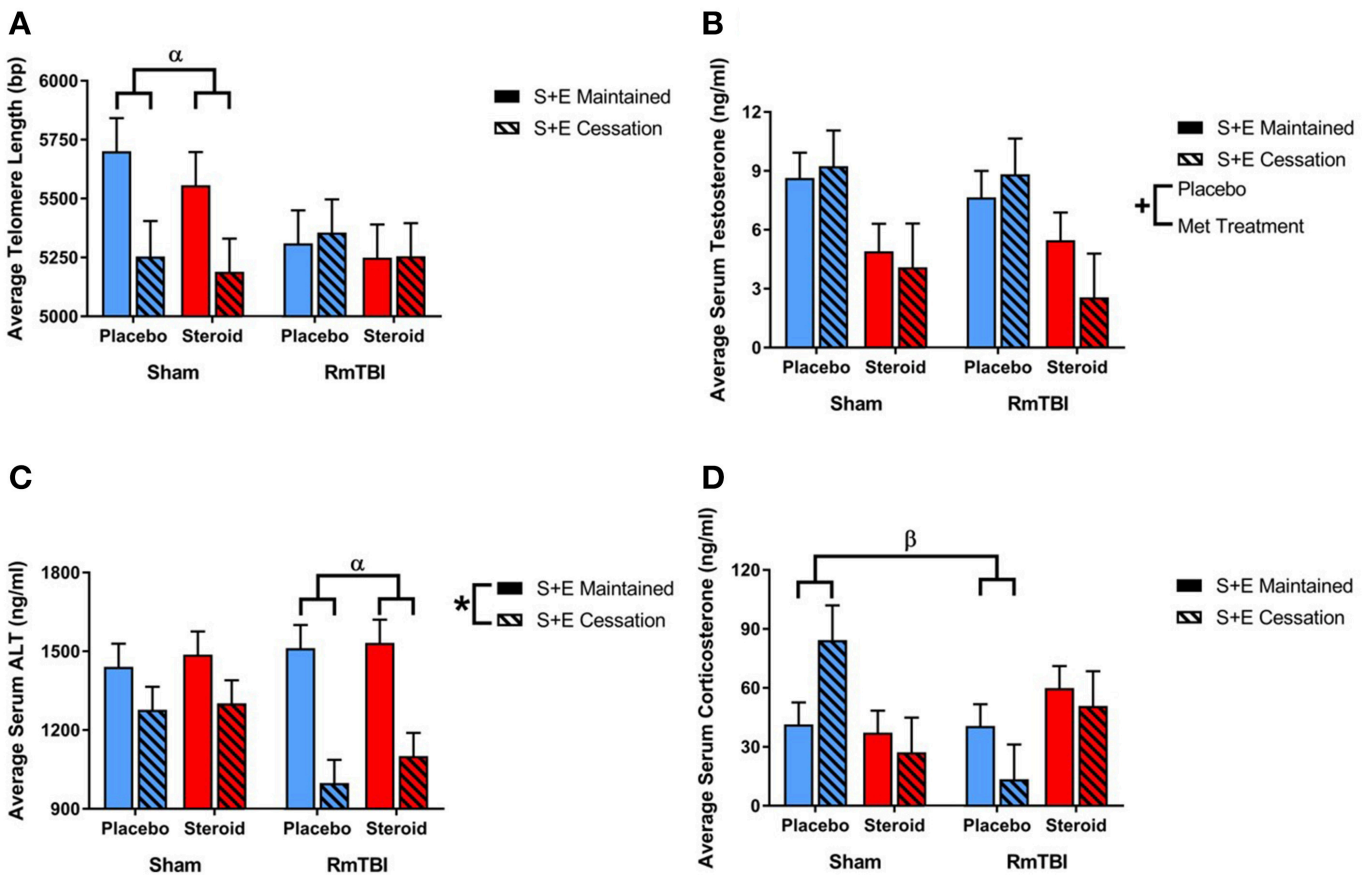
Bar graphs displaying the results from telomere length analysis and serum ELISA results. Means ± standard error are displayed where ^*^main effect for SEC, ^+^main effect of Met treatment, ^α^significant RmTBI by SEC interaction, and ^β^significant Met treatment by RmTBI interaction *p* ≤ 0.05. **(A)** Displays average telomere length for each of the groups at time of sacrifice. *Post-hoc* analysis demonstrated that the Injury by SEC interaction was driven by the sham animals, whereby telomere length was significantly reduced in response to cessation of AAS and exercise if the rat did not receive repetitive injuries, but was not reduced in animals that experienced cessation of AAS and exercise, and RmTBI, *p* = 0.02. **(B)** Displays the mean serum levels of testosterone at time of sacrifice. **(C)** Displays mean serum levels of ALT at time of sacrifice. *Post-hoc* analysis demonstrated that the Injury by SEC interaction was driven by animals in the RmTBI group, whereby those that experienced cessation of AAS and exercise exhibited significant decreases in ALT levels as compared to animals with RmTBI that maintained treatment throughout the experiment, *p* < 0.01. **(D)** Displays mean serum levels of corticosterone at time of sacrifice. *Post-hoc* analysis showed that the Met Treatment by Injury interaction was driven by the placebo group, where sham animals demonstrated elevated corticosterone and animals in the placebo group that received RmTBIs exhibited reductions in corticosterone, *p* = 0.02. RmTBI, repetitive mild traumatic brain injury; S+E, steroid and exercise; ELISA, enzyme-linked immunosorbent assay; ALT, alanine aminotransferase.

Ear notch skin samples were examined at PID15 for changes in telomere length. There was a significant RmTBI by SEC interaction [*F*_(1, 62)_ = 4.67, *p* = 0.04]. *Post-hoc* analysis demonstrated that the Injury by SEC interaction was driven by the sham animals, whereby telomere length was significantly reduced in response to cessation of AAS and exercise if the rat did not receive repetitive injuries, but was not reduced in animals that experienced cessation of AAS and exercise, and RmTBI, *p* = 0.02. There were no other significant main effects or interactions. See Figure 6.

### Serum Biomarkers

Serum levels of testosterone, corticosterone, and alanine aminotransferase (ALT) were examined at PID15. The three-way ANOVA for testosterone demonstrated a main effect of Met treatment, [*F*_(1, 62)_ = 12.53, *p* < 0.01], but not of RmTBI, and SEC, nor were there any significant interactios between RmTBI, Met treatment, or SEC.

The three-way ANOVA for corticosterone failed to show any significant main effects of RmTBI, Met treatment, or SEC, however there was a significant Met treatment by Injury interaction, [*F*_(1, 55)_ = 7.92, *p* < 0.01]. *Post-hoc* analysis showed that the Met Treatment by Injury interaction was driven by the placebo group, where sham animals demonstrated elevated corticosterone and animals in the placebo group that received RmTBIs exhibited reductions in corticosterone, *p* = 0.02.

The three-way ANOVA for ALT revealed a main effect of SEC **[***F*_(1, 31)_ = 26.86, *p* < 0.01], but not of RmTBI or treatment. However, there was a significant Injury by SEC interaction, [*F*_(1, 31)_ = 0.60, *p* = 0.03]. *Post-hoc* analysis demonstrated that the Injury by SEC interaction was driven by animals in the RmTBI group, whereby those that experienced cessation of AAS and exercise exhibited significant decreases in ALT levels as compared to animals with RmTBI that maintained treatment throughout the experiment, *p* < 0.01. See Figure 6.

## Discussion

During adolescence, RmTBI, AAS use, and AAS withdrawal are all associated with changes in anxiety, depression, mood irritability, aggression, and cognitive function (5, 17, 23). As AAS-withdrawal symptoms overlap with that of PCS, we hypothesized that abrupt steroid and exercise cessation after RmTBI would potentiate behavioral impairments associated with PCS. In support of our hypothesis, SEC prolonged post-injury loss of consciousness and exacerbated, anxiety-like behaviors and short-term working memory. SEC also increased general activity in the open field and weight gain in both sham and RmTBI rats. However, these changes may have resulted from the absence of the running wheels inaccessibility, producing a need to exercise in addition to a calorie surplus induced weight gain (57, 58). Consistent with previous studies (59, 60) RmTBI alone produced loss of consciousness, motor and balance deficits, increases in anxiety-like behavior, and working memory deficits. Also, in line with previous findings, Met treatment produced increases in depressive-like behavior (61, 62). Contrary to prior studies however, we failed to identify an increase in aggression in our Met treated rats (19, 63–65). Interestingly, when Met treatment was combined with RmTBI, we actually found a reduction in win percentage in the dominance tube, suggesting that that Met may affect the neural circuitry involved in aggression in a different manner than other commonly used AAS.

In addition to their effects on behavior, RmTBI and SEC interacted to induce changes in telomere length (TL). Previous findings in our laboratory have demonstrated mTBI induced reductions in TL (66), and shortened TL has been implicated in neurodegenerative diseases and age-related cognitive decline (67, 68). Telomeres are tandemly repeating, non-coding sequences of DNA capping the ends of eukaryotic chromosomes which are vulnerable to shortening, especially in neurons, as telomerase and other neuroprotective peptides are affected by oxidative stress (69, 70). The findings from the present study are therefore quite interesting. The SEC-sham animals had relatively similar TL to the RmTBI, suggesting that abruptly quitting Met in adolescence may have detrimental effects on TL, independent of injury. This reduction in TL may be in response to the sudden drop in androgen levels, as androgens have been shown to increase telomerase function (71), or could be associated with the absence of exercise, as exercise has also been shown to increase TL (59). Given that TL has been used as a gauge of neuronal aging and health (72), the SEC-induced shortening in TL further contributes to growing literature that AAS can potentiate neuronal impairments (17).

Typically, chronic AAS users experience a suppression of the hypothalamic-pituitary-gonadal (HPG) axis as a result of negative feedback inhibition, reducing the amount of naturally produced testosterone in circulation (73, 74). Met treatment was found to lower serum testosterone levels, verifying that in our study Met treatment did suppress the HPG axis. In addition, we also examined circulating corticosterone levels as previous literature suggested that AAS can alter the innate stress response, increasing circulation of glucocorticoids (75), while AAS withdrawal symptoms may be associated with changes in glucocorticoid action (23). Surprisingly, there were no effects of SEC on circulating levels of corticosterone, however there was an interaction between RmTBI and Met treatment, whereby RmTBI rats exposed to Met had increased corticosterone compared to the Met-exposed sham groups. This increase in corticosterone following RmTBI and Met treatment is likely due to injury- and AAS-induced dysfunction of the HPA axis; the HPA axis is vulnerable to increased androgen circulation (76), and RmTBI (43, 77). However, given that pediatric mTBI may lower cortisol levels (22), Met may be compensating for this loss by escalating the glucocorticoid response in adolescence and ironically providing neuroprotection to the injured brain. Finally, with Met being an orally consumed AAS, which has been shown to induce hepatotoxic effects through first pass metabolism (14, 18), we sought to examine the effects of Met treatment, SEC, and RmTBI on liver function by measuring ALT levels, a known biomarker of liver damage (78). SEC was found to decrease circulating levels of ALT in both RmTBI and sham animals, but the SEC-induced reduction of ALT was significantly greater in the RmTBI group. Given that animals in the SEC group had abstained from Met consumption for ~14 days prior to sacrifice, it is not surprising that they had lower ALT levels as the hepatotoxic compounds would have been removed from their systems. However, as the SEC also lowered ALT in the placebo groups, it is possible that ALT levels were more representative of exercise exposure than Met treatment, as wheel running in rodents has been shown to increase ALT (79). In addition, the intensified reductions in ALT levels we identified in the RmTBI + SEC groups, suggests that RmTBI influenced general liver health, and warrants future investigations. Overall, our results of circulating corticosterone and ALT demonstrate that Met exposure in adolescence can exacerbate the systemic response to injury and can significantly impact an individual's health.

As the PFC plays an important role in executive function, cognition (80), personality, and social behavior (81), it is particularly vulnerable to the negative consequences associated with AAS (61) and RmTBI (59). Given that the PFC matures much slower than other brain regions, undergoing significant development in adolescence, alterations to mRNA expression and protein translation may consequentially affect healthy neurodevelopment (82). In the present study mRNA expression in the PFC was examined in the context of Met treatment, SEC, and RmTBI. *Bdnf* plays a role in the developing PFC and is involved in brain plasticity related processes. Although not found in this study, AAS use has been shown to decrease *Bdnf* expression (61), which has been linked to abnormal social behavior (83). Therefore, SEC may have had positive effects in the PFC, as it increased levels of *Bdnf* expression. *Maoa* is involved in serotonin metabolism, and alterations in expression are associated with maladaptive behavioral responses to stress, such as depressive-like behaviors (47, 48). This increase in Maoa expression due to Met treatment and SEC would be consistent with our finding that Met promotes depressive-like behavior, in-line with existing literature on the depressive effects of AAS (62, 84). Complimentary to this finding, we also showed that *Iba1*, a known marker of microglial activation (45), exhibited increased expression in Met + SEC animals. Augmented inflammation in the PFC may contribute to the cognitive and withdrawal symptoms typically identified in AAS-users (85). *GR* expression was also increased due to Met treatment; possibly because androgens inhibit glucocorticoid binding to GRs (86, 87), or because Met treatment increased circulation of corticosterone. Nonetheless, increased GR activation is associated with alterations to the stress response, which could be responsible for increased neurotoxicity and further exacerbation of cognitive impairments (43, 44). We were able to show that Met treatment and SEC produced mRNA changes in the PFC that potentially contribute to stress, and in turn affect depressive-like behavior.

The AMYG is a brain region that is particularly vulnerable to both RmTBI and AAS action as it is heavily implicated in the stress response and emotional regulation (88, 89). Changes in gene expression in the AMYG can influence neuronal excitability and produce anxiogenic and depressive effects that may contribute to anxiety and mood-related disorders (90). SEC had substantial effects on gene expression in the AMYG. *Maoa* levels were affected, suggesting that serotonin levels are also altered in the AMYG, coinciding with previous literature demonstrating that AAS use may produce persistent changes to serotonergic circuitry after drug cessation in adolescence (64). In addition, increased expression of *Iba1* suggests a state of oxidative stress and neuroinflammation in the AMYG (45, 91). *GR* expression was also increased, but those with RmTBIs exhibited less of an increase, possibly because Met treatment upregulated *GR* expression before treatment stopped. Interestingly *Bdnf* expression in the AMYG was influenced by an interaction between RmTBI, Met, and SEC, which warrants further investigating as altered *Bdnf* levels in the AMYG have been implicated in depression (92). The mRNA expression changes suggest that SEC lead to increased neuroinflammatory and stress responses in the AMYG, which could interact with many brain regions to produce anxiogenic and depressive effects, while feeding into a persistent cycle of systemic stress.

Conversely, the HPC was the least affected by SEC and Met treatment. The HPC, like the AMYG, is part of the limbic system, and is in part responsible for emotional regulation (93). However, it also plays a role in cognitive function, memory consolidation, and a key site for adult neurogenesis (94). SEC increased *Iba1* expression in the HPC which as a marker of microglial activity (45), is associated with the suppression of neurogenesis in the HPC (95), contributing to symptoms of depression (96) which are prevalent in AAS-withdrawal (18). Conversely, increased GR activation has been associated with reduced neurogenesis and HPC damage (97). *GR* expression was increased by Met treatment, likely due to the altered stress response that AAS can enact on the brain (61) as well as an attempt by the HPC to lower HPA function through negative feedback mechanisms.

Lastly, the PIT is a neuroendocrine structure, implicated in the regulation of many physiological processes including growth, reproduction, and stress (98). Notably, the PIT plays a large role in the HPA axis, receiving input from the hypothalamus and subsequently leading to the downstream activation of glucocorticoid synthesis and secretion (99). Because of its involvement in the brain's response to stress and hormonal function, it is susceptible to permutation from RmTBI and the effects of AAS-withdrawal. Given that we identified changes in anxiety and depressive-like behaviors, in addition to serum markers of stress, we examined mRNA expression in the PIT. Like the PFC and AMYG, SEC had profound effects on gene expression. Firstly, *Maoa* expression was increased, potentially affecting the HPA axis, and downstream ACTH secretion (100). Previous findings have shown that treatment with selective serotonin reuptake inhibitors (SSRIs) have increased HPA activation (101) and that general stimulation of serotonergic circuitry surrounding the HPA axis has led to increased HPA activation (102), therefore *Maoa* upregulation could be a function of increased serotonergic activity in the PIT. Given that *ER* is involved in the transcriptional control of endocrine functions such as the synthesis and release of growth hormone, prolacatin, and gonadotropin hormones (103, 104), alteration in expression levels of this gene due to SEC and Met treatment could have significant consequences during the neurodevelopmentally sensitive period of adolescence. *ER* can be greatly affected by AAS too since the excess androgens can aromatize in the brain to estradiols which bind to estrogen receptors, producing both genomic and non-genomic effects (14, 53). *Creb* was affected by SEC in the PIT, however there were also interactions between Met and SEC, and Met and RmTBI. These peculiar results likely reflect the integral role that *Creb* plays for healthy development of the PIT (105). Alterations to *Creb* levels through Met, SEC, or RmTBI could prove detrimental given the PIT is very sensitive to *Creb* levels, as seen in pituitary tumors or pituitary hypoplasia (105). Like the other 3 brain regions examined, SEC increased *Iba1* mRNA expression. The HPA axis has bidirectional communication with the neuroimmune system (76), and the release of pro-inflammatory cytokines due to microglial reactivity (106) affects PIT development and enhances HPA axis function through increased ACTH production (106). Finally, we saw increased *GR* expression due to Met exposure. Typically, acute HPA activation results in the release of glucocorticoids as a protective response (107) which also play a regulatory role by way of negative feedback inhibition on the PIT (108). However, AAS have been shown to alter behavior through increased glucocorticoid signaling (109) manifesting as depression and anxiety-like behaviors consistent with our behavioral findings. These effects can be further exacerbated in adolescence as previous findings have shown adolescent animals to have longer HPA responses than adults (97).

In summary, this study identified negative and alarming outcomes associated with RmTBI and SEC in adolescence. Behaviourally, we identified cumulative effects of RmTBI and SEC to loss of consciousness, anxiety-like behavior, and short-term working memory, in addition to changes in aggression and depressive-like behavior due to Met treatment. Interestingly, we saw even more robust effects of Met treatment and SEC in mRNA expression changes across 4 different brain regions, most of which demonstrated alterations to serotonergic circuitry, neuroinflammation, and an enhanced stress response. Interestingly, although serum corticosterone levels did not reflect any change due to SEC, Met was able to increase the glucocorticoid response to RmTBI. Opposite to our findings of lower corticosterone after RmTBI, studies of severe TBI show acute increases in cortisol after injury in both children (110) and adult patients (111, 112). As cortisol levels can fluctuate depending on TBI severity, there is a need for more research on the beneficial effects of AAS on glucocorticoid secretion since previous clinical trials have found corticosterone treatments for TBI to increase mortality rates (113). To date, only 2 studies have tried to investigate the interactions between AAS abuse and mTBI with varying results (114, 115), leaving a gap in the literature on a growing problem among both adolescent and adult athletes.

Although there are numerous strengths to this article, it is not without it's limitations. For example, future studies should examine the effects of SEC on female rats; while the usage of AAS in female adolescents is well-below that of males, their lifetime prevalence is still significant (13, 26, 27). In addition, mRNA was extracted from tissue in the specified brain regions, but this was not cell specific and we were unable to attribute mRNA changes to neurons or surrounding glial cells. While an ideal study would have examined cell specific gene expression changes, protein levels, and immunohistochemistry, this would have required a substantially larger sample size, but leaves ample opportunity for future discoveries in the field. However, to our knowledge, this is also the first study to examine the affects of sudden withdrawal of AAS has on the neurological function of athletes who have sustained multiple concussion. Given the findings from this study, we believe that future research examining the affects of SEC post-injury are warranted and suggest that clinical studies be done to confirm if athletes should taper the cessation of their AAS regime rather than quit cold turkey in an effort to minimize negative outcomes. Additionally, Met's ability to promote glucocorticoid secretion after injury opens potential therapeutic avenues of exogenous hormone administration for clinical treatment of mTBI.

## Ethics Statement

All reported experiments were carried out in accordance with the Canadian Council of Animal Care and received approval from the University of Calgary Conjoint Faculties Research Ethics Approval Board.

## Author Contributions

JT was involved in the experimental design, data collection, and writing of the manuscript. RC was involved in data collection and writing of the manuscript. CD was involved in experimental design and manuscript writing. SS was involved in experimental design and manuscript writing. RM was involved with experimental design, data collection, data analysis, and writing of the manuscript.

### Conflict of Interest Statement

The authors declare that the research was conducted in the absence of any commercial or financial relationships that could be construed as a potential conflict of interest.
